# Reprogramming of Mesothelial-Mesenchymal Transition in Chronic Peritoneal Diseases by Estrogen Receptor Modulation and TGF-β1 Inhibition

**DOI:** 10.3390/ijms21114158

**Published:** 2020-06-10

**Authors:** Robert B. Wilson, Rami Archid, Marc A. Reymond

**Affiliations:** 1 Department of Upper Gastrointestinal Surgery, UNSW, Liverpool Hospital, Liverpool NSW 2170, Australia; 2 Department of General and Transplant Surgery, National Center for Pleura and Peritoneum, University Hospital Tübingen, 72076 Tübingen, Germany; Rami.Archid@med.uni-tuebingen.de (R.A.); Marc.Reymond@med.uni-tuebingen.de (M.A.R.)

**Keywords:** EMT, MMT, TGF-β1, tamoxifen, estrogen receptor, peritoneal metastasis, encapsulating peritoneal sclerosis, peritoneal dialysis, HIF-1α, Cancer associated fibroblast, Src

## Abstract

In chronic peritoneal diseases, mesothelial-mesenchymal transition is determined by cues from the extracellular environment rather than just the cellular genome. The transformation of peritoneal mesothelial cells and other host cells into myofibroblasts is mediated by cell membrane receptors, Transforming Growth Factor β1 (TGF-β1), Src and Hypoxia-inducible factor (HIF). This article provides a narrative review of the reprogramming of mesothelial mesenchymal transition in chronic peritoneal diseases, drawing on the similarities in pathophysiology between encapsulating peritoneal sclerosis and peritoneal metastasis, with a particular focus on TGF-β1 signaling and estrogen receptor modulators. Estrogen receptors act at the cell membrane/cytosol as tyrosine kinases that can phosphorylate Src, in a similar way to other receptor tyrosine kinases; or can activate the estrogen response element via nuclear translocation. Tamoxifen can modulate estrogen membrane receptors, and has been shown to be a potent inhibitor of mesothelial-mesenchymal transition (MMT), peritoneal mesothelial cell migration, stromal fibrosis, and neoangiogenesis in the treatment of encapsulating peritoneal sclerosis, with a known side effect and safety profile. The ability of tamoxifen to inhibit the transduction pathways of TGF-β1 and HIF and achieve a quiescent peritoneal stroma makes it a potential candidate for use in cancer treatments. This is relevant to tumors that spread to the peritoneum, particularly those with mesenchymal phenotypes, such as colorectal CMS4 and MSS/EMT gastric cancers, and pancreatic cancer with its desmoplastic stroma. Morphological changes observed during mesothelial mesenchymal transition can be treated with estrogen receptor modulation and TGF-β1 inhibition, which may enable the regression of encapsulating peritoneal sclerosis and peritoneal metastasis.

## 1. Introduction

The coelomic microenvironment influences the behaviour of peritoneal mesothelial cells (PMC) and stromal recruitment, and is linked to mesothelial-mesenchymal transition (MMT). MMT is defined as the peritoneum-specific process of epithelial-mesenchymal transition (EMT) in chronic peritoneal diseases. These include encapsulating peritoneal sclerosis (EPS), invasive endometriosis (EM) and peritoneal metastasis (PM). Peritoneal MMT involves the transformation of PMC, which normally has a stable basement membrane/extracellular matrix (ECM) attachment and epithelial-like phenotype, into fibroblast-like cells with invasive ability and mesenchymal phenotype. 

**Figure 1:**
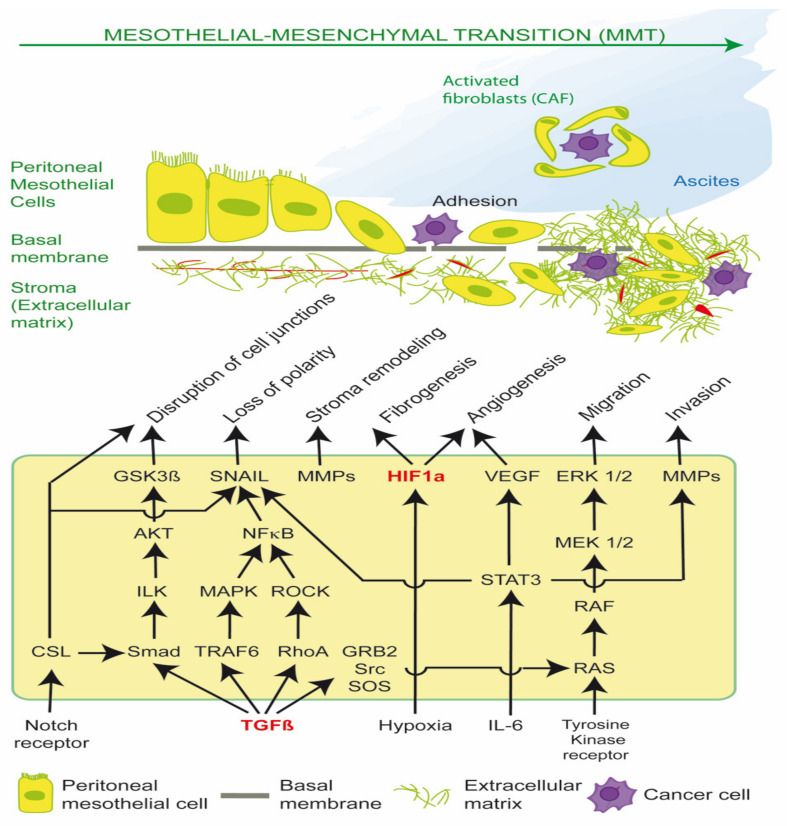
Transforming Growth Factor β1 (TGF-β1) and Hypoxia-inducible factor (HIF) signaling pathways in EPS and PM. Activation of these pathways results in mesothelial-mesenchymal transition (MMT) of peritoneal mesothelial cells into activated fibroblasts in encapsulating peritoneal sclerosis (EPS), and cancer associated fibroblasts (CAFs) in peritoneal metastasis (PM). CAFs have reciprocal receptor tyrosine kinase activation and metabolic coupling with cancer cells in the tumor microenvironment. CAFs form tumor spheroids with epithelial cancer cells, promote ascites and transcoelomic metastasis, and facilitate basement membrane/extracellular matrix invasion.

MMT is mediated principally by Transforming Growth Factor β1 (TGF-β1) and Hypoxia-inducible factor (HIF), which drive cancer stromal recruitment, myofibroblast or cancer associated fibroblast (CAF) formation, angiogenesis, aerobic glycolysis, metabolic coupling, ascites, peritoneal fibrosis and cell migration (Figure 1) [1,2,3,4,5,6,7,8]. Myofibroblasts and CAFs produce a desmoplastic stroma due to their synthesis of ECM proteins, such as collagen type I and III, glycosaminoglycans and proteoglycans, fibronectin, α-smooth muscle actin (α-SMA), tenascin C (TNC) and hyaluronan. A dense stroma is associated with elevated tumor hydrostatic pressures, tumor stiffness, hypoxia and resistance to chemotherapy and immunotherapy [1,9]. CAFs can also remodel the ECM, align fibrils and provide safe passages along which cancer cells can travel, enhancing cancer cell migration [3]. 

This article aims to provide a narrative review of the treatment of MMT in chronic peritoneal diseases, drawing on the similarities in EPS and PM pathophysiology [1] and focusing in particular on estrogen receptor (ER) modulators, the inhibition of TGF-β1 pathways and stromal reprogramming.

## 2. TGF-β1, Src and MMT

The observation by Peyton Rous in 1909 that a spindle cell sarcoma in chickens was caused by a transmissible virus (Rous sarcoma virus) led to the discovery of *c-src*, the first human proto-oncogene [10]. The protein product of *c-src* is Src, a membrane associated non-receptor tyrosine kinase. Src regulates cell proliferation, differentiation, transformation, anoikis resistance, invasion, migration, and survival. Src is required for the phosphorylation of TβR-II, which activates TGF-β1 pathways. Bone morphogenetic proteins (BMP) or TGFβ ligands (TGF-β1) bind the TGFβ receptor II (TβR-II), which recruits and phosphorylates TGFβ receptor I (TβR-I). TGF-β1 plays a critical role in epithelial-mesenchymal transition (EMT) and mesothelial-mesenchymal transition (MMT) via canonical SMAD 2/3 signaling and non-canonical RAS/RAF/MEK/ERK pathways; the PI3K/AKT/mTOR pathway; and the signal transducer and activator of transcription 3 (STAT3) pathway, which regulates the expression of c-Myc and Cyclin D1. The pioneering work of Dr Rous led to the discovery of receptor tyrosine kinases (RTK) including c-Kit, VEGFR, PDGFR, EGFR, FGFR and IGFR, which also activate Src; and specific RTK inhibitors (imatinib, sunitinib, sorafenib) and Src inhibitors (dasatanib, bosutinib) [10,11,12,13]. TGF-β1 induced EMT programs have been shown to inhibit estrogen receptor alpha (ER-α) nuclear translocation and promote cytoplasmic retention of ER-α, with increased physical ER-α interactions with Src, EGFR and IGFR and activation of MAP kinases (ERK1/2 and p38 MAPK) [14]. 

### 2.1. Cellular Homeostasis, Cytoplasmic Signaling and Glycolysis

Otto Warburg originally hypothesized that cancer was a mitochondrial metabolic disease, and switching cellular energy production from mitochondrial oxidative phosphorylation to cytosolic glycolysis was sufficient to promote carcinogenesis [15]. The stabilization of HIF-1α in the presence of TGF-β1 signaling, iron deficiency, mitochondrial dysfunction, hypoxia or oxidative stress enables the activation of the hypoxia response element (HRE). The HRE upregulates glycolytic enzymes and lactate dehydrogenase (LDH) to maintain the rapid production of ATP via conversion of pyruvate to lactate. HIF and oncogenic tyrosine kinases (FGFR1) promote pyruvate dehydrogenase kinase (PDHK1) inhibition of PDH in the mitochondria. This prevents pyruvate being converted to acetyl-CoA and used in oxidative phosphorylation. The glycolytic switch which occurs under cellular normoxia is known as the Warburg effect, which minimizes the production of reactive oxygen species (ROS) in mitochondria and enables cells to maintain ATP production and evade caspase and mitochondrial mediated apoptosis [1,2,3,4,5,6,7,8]. Under cellular normoxia, the transcriptional activation of HIF-1α by hydrogen peroxide, superoxides, thrombin and NADPH oxidase 4 (NOX4) is upregulated by the nuclear factor kappa light chain enhancer of activated B cells (NF-κB) [16]. The ability of cells to detach from the basement membrane, resist anoikis and acquire migratory ability and mesenchymal phenotypical properties via cytosolic glycolysis, glycation, lactate production, extracellular acidosis, actin re-arrangement and lamellipodia formation is now recognized as a key process in PM and EPS [1,17]. 

The Lucké frog renal carcinoma project showed that normal cytoplasmic signaling was able to control the fate of cells, even when they possessed a malignant genome [18]. Under normal homeostatic conditions, signaling via canonical TGF-β1 pathways results in tumor suppression. However, under the influence of sustained or extreme tissue damage, damage associated molecular patterns (DAMPs), pathogen associated molecular patterns (PAMPs), high-mobility group box 1 protein (HMGB1), cytokine, heat shock protein (HSP) or NF-κB release, oxidative stress, hypoxia, increased glycolysis, dicarbonyl stress, extracellular acidosis or chronic inflammation, TGF-β1 acts as a promoter of activated fibroblasts (myofibroblasts) and tumors via aberrant cytoplasmic and transmembrane signaling. This is known as the *TGF paradox* [19,20,21,22,23,24,25,26,27,28,29,30,31]. 

Failure to turn *off* myofibroblasts in a wound leads to chronic fibrosis or CAF induced cancer invasion. Recently, it was shown that the twist basic helix loop helix transcription factor 1 (TWIST1)- Paired Related Homeobox 1 (Prrx1)-TNC positive feedback loop can be permanently switched *on*, leading to the irreversible activation of fibroblasts under pathological conditions including inflammation, infection, and tumorigenesis [30]. This may explain the similarities in the pathogenesis of EPS and peritoneal metastasis. The concept of environmental cues controlling cell fate was further elucidated in a recent study, which showed the destiny of normal adult intestinal cells, including the trans-differentiation into stem cells, was determined not by their genome, but by signals from the cells’ surroundings [32]. 

### 2.2. Role of DAMP Receptors and NF-κB

The activation of NF-κB regulates almost all the gene products of inflammation (IL-1, IL-6, COX-2, 5-lipoxygenase), as well as proliferation, anti-apoptosis, angiogenesis, invasion and metastasis genes (VEGF, adhesion molecules, TWIST, C-X-C chemokine receptor type 4 (CXCR4), matrix metalloproteases (MMP)) [33]. Peritoneal injury, tumor necrosis factor-α (TNF-α), cellular oxidative stress or cell necrosis result in the extracellular release of nuclear high mobility group box 1 (HMGB1) protein and the activation of NF-κB. This involves pattern recognition receptors (PRRs), which are receptors for DAMPs and PAMPs. Normally, DAMPs are sequestered in the intracellular compartment or ECM and not exposed to recognition by the innate immune system [26,32]. Some examples of DAMPs include HMGB1, mitochondrial DNA, TNC [30,32], ROS, NF-κB, advanced glycation endproducts (AGEs), low density lipoprotein, fibrinogen, heat shock proteins (HSP), S100A8/A9, fibronectin, heparan sulphate and LMW oligomeric hyaluronan fragments. PAMPs are particularly important in EPS initiated by recurrent bacterial or fungal chronic ambulatory peritoneal dialysis (CAPD) peritonitis. Examples of PAMPs are pathogen structural molecules, such as bacterial flagellin, peptidoglycan, and lipopolysaccharides (LPS); oomycete glucans, and fungal chitin [34]. 

DAMPs and PAMPs are recognized by PRRs borne by cells of the innate immune system. These include dendritic cells, macrophages and leukocytes, but also peritoneal mesothelial cells, fibroblasts and endothelial cells. The activation of PRRs initiate and maintain inflammatory and fibrogenic pathways, particularly via non canonical TGF-β1 signaling [20,26,32]. Examples of PRRs include Toll-like receptors (TLRs), receptors for advanced glycation end-products (RAGE), nucleotide-binding oligomerization domain (NOD)-like receptors (NLRs), C-type lectin receptors (CLRs), retinoic acid inducible gene-I (RIG-I)-like receptors (RLRs), and DNA sensors, including absent in melanoma 2 protein (AIM2)-like receptors (ALRs) [27,32]. 

### 2.3. Role of HMGB1

Nuclear HMGB1 is a transcriptional cofactor of p53, p73, retinoblastoma protein, NF-κB, and nuclear hormone receptors, including the estrogen receptor. It normally acts as a tumor suppressor by maintaining chromosomal stability and limiting pro-inflammatory nucleosome release and activity. However, HMGB1 can be translocated from the nucleus to the cytoplasm and secreted by immune cells or extruded by transformed, necrotic or injured cells, and thus act as a DAMP [18,24]. Cytoplasmic translocation of HMGB1 and subsequent extracellular release on necrotic cell death can be facilitated by activated PARP [18,28]. Extracellular HMGB1 has cross ligand binding for RAGE and TLR-2 and -4 [35]. Cancer associated fibroblasts (CAFs) have been shown to secrete HMGB1 via autophagy, which activates TLR-4 receptors expressed by cancer cells to increase the stem cell phenotype and tumor growth [18,31]. 

The activation of PRRs by DAMPS or PAMPS [18,24] initiates a cascade of inflammatory and proliferative events, mediated by NF-κB, cytokine and TGF-β1 release and IL-1 receptor-associated kinase signaling (IRAK) [18,23]. This leads to downstream activation of EMT transcription factors (SNAIL, TWIST, ZEB), suppression of E-cadherin, and the promotion of EMT and MMT. 

Src is required for the phosphorylation of TβR-II, which activates TGF-β1. TGF-β1 plays a critical role in epithelial-mesenchymal transition (EMT) and mesothelial-mesenchymal transition (MMT) via canonical SMAD signaling and non-canonical RAS/RAF/MEK/ERK pathways; the PI3K/AKT/mTOR pathway; and the STAT3 pathway, which regulate the expression of SNAIL, c-Myc and Cyclin D1. Such pathways can be stimulated by extracellular cues, including tissue damage, acidosis, reactive oxygen species (ROS), hypoxia, cytokines, and DAMPS, leading to *redox fibrosis.* Both the TGF-β1-induced SMAD-dependent and SMAD independent pathways converge on the activation of SNAIL. SMAD is the homologue of Drosophila protein MAD (mothers against decapentaplegic) and the *Caenorhabditis elegans* protein SMA (small body size). The loss or suppression of E-cadherin by EMT transcription factors such as SNAIL leads to the re-organization of actin cytoskeleton, loss of cell polarity, disruption of cell to cell adherens junctions, and the detachment of cells from the basement membrane. Stromal remodeling is mediated by matrix metalloproteases (MMP), which enhances cell migration through the ECM and further release of latent TGF-β1 and VEGF. The activation of HIF occurs via hypoxia, oxidative stress and mechanoreceptor transduction and enhances production of ECM proteins (fibrogenesis) and VEGF (angiogenesis). The sustained release of TGF-β1, ROS and IL-6 leads to transformation of peritoneal mesothelial cells into activated fibroblasts (myofibroblasts), and in the case of PM, cancer associated fibroblasts (CAFS). CAFs support cancer cell survival by providing nutrients (glutamine, lactate); acidifying the extracellular space; promoting cancer spheroids and ascites; and preventing host T cell immunosurveillance. The disruption of the peritoneal glycocalyx and cell integrins and exposure of the basement membrane enable peritoneal metastasis, by promoting cancer cell adherence to the peritoneal surface and subsequent invasion and proliferation (Figure 1) [1].

## 3. Generation of Activated Fibroblasts via MMT

Normal PMC, but also other host cells (endothelial cells, pericytes, resident fibroblasts, bone marrow derived mesenchymal stem cells, adipocytes), can be transformed via MMT into glycolytic activated fibroblasts (CAFs). This is mediated by TGF-β1 release, k-ras and p53 expression, cancer cell secretion of exosomal hydrogen peroxide and IL-6, loss of caveolin-1 and induction of CAF oxidative stress, HIF-1α, NF-κB and autophagy [1,9,30,36]. The result of such a transformation is tumor progression with dense stroma, metabolic coupling between cancer cells and CAFs, and chemotherapy and immunotherapy resistance. Caveolin-1 negative, glycolytic fibroblasts dramatically promote tumor growth with a ∼4-fold increase in tumor mass and ∼8-fold increase in tumor volume [6]. CAFs have a stable genome, are chemotherapy tolerant and orchestrate the tumor microenvironment (TME) by ‘cross talk’ with other stromal cells and epithelial cancer cells [1,2,3,4,5,6,7,8,9,30]. Such ‘cross talk’ between CAFs and cancer cells when in close proximity includes the reciprocal activation of multiple RTKs (FGF/FGFR, EGF/EGFR, ephrin B1/EPHA2) and the phosphorylation of CAF PDGFRA, IGF1R and multiple cyclin dependent kinase (CDK) proteins [7]. CAFs secrete inflammatory cytokines, chemokines and growth factors into the TME, which promote cancer cell ‘stemness’ and migration and the polarization of M2-like, PD-1 positive tumor associated macrophages (TAMs) [36,37]. This includes autocrine and paracrine effects of secreted IL-6, IL-8 (CXCL-8), VEGF, prostaglandin-E2, CXCL-1, C–C motif chemokine ligand 2 (CCL2) or monocyte chemotactic protein-1 (MCP-1), stromal cell-derived factor-1 (SDF-1) or CXCL-12, hepatocyte growth factor (HGF), and TGF-β1 [1,36]. TGF-β1 in the TME can be derived from epithelial cancer cells, CAFs, activated platelet degranulation or from latent TGF-β1 sequestered in the ECM [1,36]. 

The 2015 consensus molecular subtype (CMS) classification of colorectal cancer [38]; and the 2015 Asian Gastric Cancer Research Group classification of gastric cancers [39] identified certain epithelial cancer subtypes with stable genomes, but with mesenchymal properties characterized by abnormal TGF-β1 signaling and a predilection for peritoneal spread and poor prognosis. These include CMS4 colon cancer and microsatellite stable/epithelial-mesenchymal transition (MSS/EMT) gastric cancer [36,38,39]. It has recently been suggested that a paracrine effect of TGF-β1 signaling related to CAFs is predominant in diffuse (MSS/EMT) gastric cancer, whilst an autocrine function of TGF-β1 may be more important in intestinal-type gastric cancer [40,41,42]. 

### 3.1. Therapeutic Approaches in MMT

**Figure 2:**
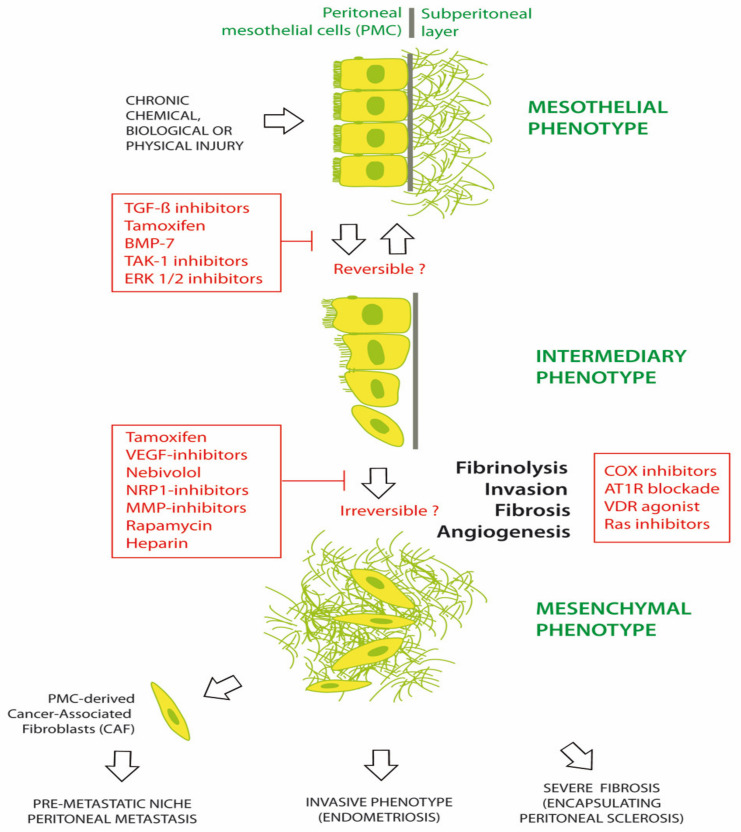
Prevention and treatment of mesothelial to mesenchymal transition (MMT).

Numerous agents have been investigated for preventing or treating MMT in chronic peritoneal diseases. Common chronic peritoneal diseases include EPS, PM and EM [1,43,44,45]. Figure 2 illustrates the progression of the peritoneum from a mesothelial to a mesenchymal phenotype, under the trigger of chemical, biological and/or physical damage. Morphological changes are initially limited with a phenotypical intermediary between epithelial and mesenchymal tissue. It is hypothesized that these changes might be reversed by early therapy. Some therapeutic agents include tamoxifen, bone morphogenetic protein-7 (BMP-7), TGF-β1 inhibitors, transforming growth factor beta-activated kinase 1 (TAK-1) inhibitor (5Z-7-oxozeaenol) and extracellular signal-regulated protein kinases 1 and 2 (ERK 1/2) inhibitors. Later in the course of disease, additional changes are observed, including fibrosis, angiogenesis, invasion of the basal membrane and loss of fibrinolysis. A range of agents, such as tamoxifen; glucocorticoids; vascular endothelial growth factor (VEGF), Neuropilin1 (NRP-1), MMP or cyclooxygenase (COX) inhibitors; mTOR inhibition (rapamycin); LMW heparin; adrenergic β1-receptor blockers (nebivolol); angiotensin I receptor (AT1-R) antagonists; and vitamin D receptor (VDR) agonists (calcitriol) have been investigated in EPS, with varying efficacy. This suggests that the profound late changes associated with MMT may be difficult to completely reverse [46,47,48,49,50]. 

### 3.2. Targeting Cancer Associated Fibroblasts

Although stromal depletion or targeting CAFs may be an attractive therapy for desmoplastic tumors, there have been unexpected consequences [51]. These include the development of invasive and undifferentiated tumors after sonic hedgehog pathway blockade, immunosuppression with ganciclovir therapy or increased toxicity with broad TGFβR blockade [52,53]. Systemic recombinant sperm hyaluronidase (PEGPH20), which degrades hyaluronan in desmoplastic pancreatic cancer stroma, and galunisertib, which blocks TGFβR1 and TGFβR2, have not been successful in clinical trials. Targeting cancer stem cells in tumors has also proven to be disappointing, with the failure of the focal adhesion kinase (FAK) inhibitor defactinib, the STAT-3 inhibitor napabucasion, the anti-NOTCH-2/3 antibody tarextumab, the anti-delta like canonical notch ligand (DLL)-4 antibody demcizumab, and the anti-DLL-3 antibody-drug conjugate rovalpituzumab tesirine [13]. Inhibiting the KRAS downstream signaling of MEK/MAPK by Selumetinib and Pimasertib, or by Rigosertib (RAS mimetic) has not been successful in the treatment of pancreatic ductal adenocarcinoma (PDAC) [13]. Thus, the concept of modulation of stromal signaling in the tumor microenvironment, by stromal cell *quiescence* rather than stromal depletion, may be a safer and more successful strategy [13,38]. Some of the advantages of re-purposing existing FDA approved drugs for use in stromal reprogramming are the potential cost savings, known safety profile and shortened drug development times [13].

### 3.3. Tamoxifen and the Peritoneum in Encapsulating Peritoneal Sclerosis (EPS)

Encapsulating peritoneal sclerosis is a chronic fibrosing condition associated with recurrent infective or chemical CAPD peritonitis, thrombin activation, fibrin and ECM deposition, cytokine and TGF-β1 release and the transformation of PMC into myofibroblasts (MMT) [54]. Submesothelial myofibroblasts derived from PMC trans-differentiation can produce up to 10 times the amount of VEGF than normal PMCs, which drives both peritoneal angiogenesis and lymphangiogenesis [45]. MMT can be caused by metabolic stress in PMC chronically exposed to bio-incompatible, hyperosmolar peritoneal dialysis fluid (PDF), containing excessive acid, lactate, glucose or glucose degradation products (GDP) [1]. Recurrent PD catheter infection leading to bacterial or fungal peritonitis, instilling PD catheters with chlorhexidine gluconate antiseptic leading to chemical peritonitis, or recurrent intra-abdominal hemorrhage are all risk factors for EPS in CAPD. Using neutral pH PDF with low GDP and minimizing high-glucose fluids with the use of icodextrin can prevent the production of advanced glycation end products (AGE), the release of TGF-β1 and the development of EPS [1]. 

**Figure 3:**
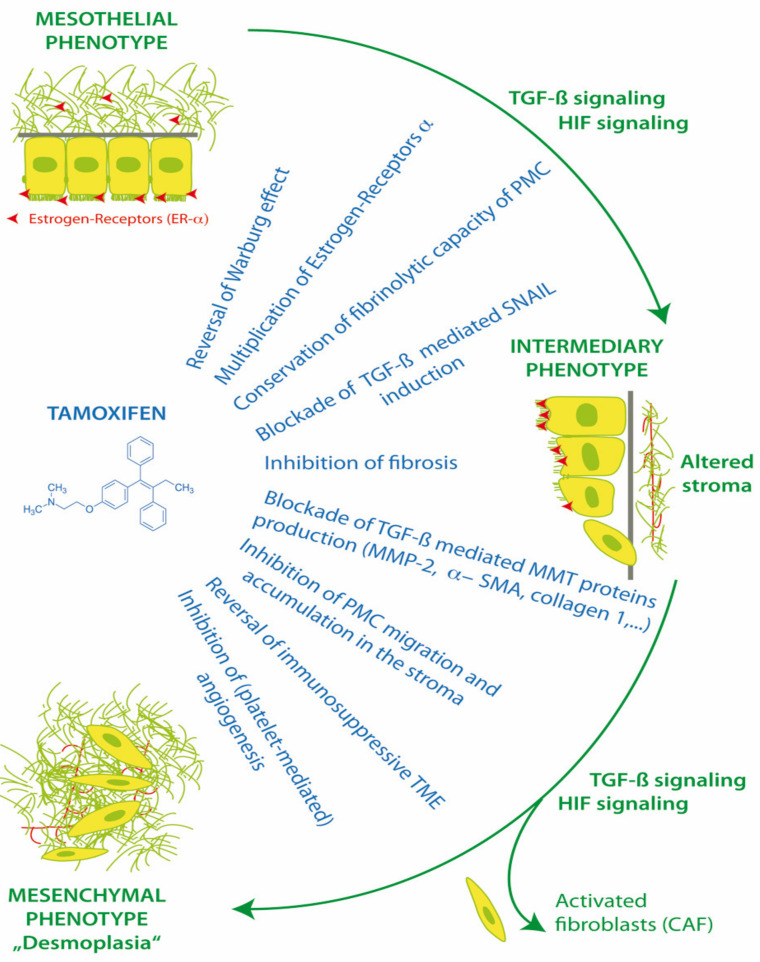
Action of tamoxifen in treatment of mesothelial to mesenchymal transition.

PMC and also stromal cells have functional membrane associated estrogen receptors (ER-α) and respond to the modulation of ER in the peritoneal microenvironment [52]. Estrogen receptors have effects via canonical nuclear translocation and transcription, but also rapid interactions with cytoplasmic pathways, including Src and TGF-β1. Thus, the activation or blockade of estrogen receptors can have a profound impact on cell fate, MMT, homeostasis, apoptosis, anoikis, peritoneal fibrosis and the progression of endometriosis and peritoneal metastasis (Figure 3).

Tamoxifen is an oral selective estrogen receptor modulator (SERM), with pleiotropic effects in different tissues. It can act as an agonist or an inhibitor of ER-α, depending on ER-α expression levels, activation function-1 (AF-1) recruitment [55], ER-α/ER-β ratios and in vivo tamoxifen metabolites. It has been shown to have utility in the therapy of chronic fibrosing conditions, including idiopathic retroperitoneal fibrosis, desmoid tumors, sclerosing cervicitis, fibrosing mediastinitis and Dupuytroen’s contracture [56]. It can inhibit the canonical TGF-β1 pathway by blocking SMAD 2/3 activation and promoting SMAD7, as well as the non-SMAD TGF-β1 pathway via the blockade of ERK1/2 MAP-kinase and the AP-1 transcription factor FRA2. Tamoxifen may thus be utilized in the therapy of TGF-β1 dependent conditions, including EPS and PM [1,47,57].

#### 3.3.1. In Vitro Experiments

In 2013, the effect of tamoxifen was evaluated in an in vitro EPS model, using normal human omental PMC. Tamoxifen blocked the TGF-β1 pathways, increased ER-α receptor expression 6-fold in PMC, and reversed the morphological changes of PMC MMT and the down regulation of E-cadherin. Tamoxifen interfered with the TGF-β1 mediated production of MMT proteins including collagen I, fibronectin, MMP-2 and α-SMA. Tamoxifen blocked the TGF-β1 induction of SNAIL transcription factor and reduced the MMT/TGF-β1 related migratory capacity of human PMCs to basal levels. Tamoxifen was able to preserve the fibrinolytic capacity of mesothelial cells exposed to TGF-β1, and return plasminogen activator inhibitor (PAI-1)/tissue plasminogen activator (tPA) ratios to basal levels [54]. 

#### 3.3.2. Animal Experiments

In the animal model, the following properties of tamoxifen were demonstrated: preservation of peritoneal mesothelium and membrane function; and potent inhibition of MMT, PMC migration, fibrosis, and neoangiogenesis [54]. The in vivo administration of tamoxifen in a mouse CAPD EPS model significantly reduced the thickness of peritoneal fibrosis and the submesothelial accumulation of PMC derived fibroblasts expressing fibroblast specific protein 1 (FSP-1). In the control mice, both MMT and the severity of peritoneal fibrosis increased during the 30-day CAPD mouse model. Tamoxifen had no effect on TGF-β1 concentrations in mouse PD effluents, but leptin levels were significantly reduced. Tamoxifen significantly reduced mouse PD submesothelial angiogenesis, peritoneal fibrosis and effluent levels of VEGF. This suggested that tamoxifen interfered with the synergism between leptin, VEGF and TGF-β1. Tamoxifen also partially preserved the peritoneal ultrafiltration capacity of CAPD treated mice. Tamoxifen provided a protective effect during both the early and late stages of peritoneal fibrosis [54].

#### 3.3.3. Clinical Findings

In the clinical setting, tamoxifen has been used since 1991, when it was first reported to have a dramatic effect and prevented mortality in two patients with retroperitoneal fibrosis. In 1992, Loureiro et al. began using tamoxifen to treat CAPD patients with EPS, and found a significant reduction in hospital admission rates, surgical abdominal complications and mortality in comparison with the conventionally treated patients (historical controls) [54]. However, the two largest subsequent clinical studies in EPS had conflicting results. The 2011 Dutch study (63 patients) reported a significantly improved mortality in the tamoxifen treated group versus controls (45.8% vs. 74.4%, *p* = 0.03) [58]. In contrast, the 2009 UK study (111 patients) showed no outcome differences in patients treated with tamoxifen, steroids, immunosuppression or combinations of these compared to control groups [59]. It was postulated that this disparity was due to less severe EPS and lower rates of renal transplantation in the UK study compared to the Dutch study [58]. Renal transplantation has been associated with accelerated EPS in patients who have previously had CAPD, particularly with calcineurin inhibitors, such as Tacrolimus and Cyclosporin, which upregulate TGF-β1. Use of m-TOR inhibitors such as Everolimus instead of calcineurin inhibitors after renal transplantation may reduce the risk of EPS [1].

Tamoxifen can reprogram stroma by inhibiting HIF and reverting transformed peritoneal mesothelial cells (PMC) from cytoplasmic glycolysis (Warburg effect) to mitochondrial respiration. Tamoxifen can up-regulate peritoneal ER-α receptors and block transforming growth factor-1 (TGF-β1), the induction of EMT transcription factor Zinc finger protein SNAI1 (SNAIL), and the suppression of E-cadherin. Tamoxifen improves peritoneal fibrinolysis and inhibits TGF-β1 induced production of extracellular matrix proteins alpha-smooth muscle actin (α-SMA), collagen type 1 and III and fibronectin. The inhibition of matrix metalloproteases (MMP-2) can decrease the ability of transformed cells to migrate through the extracellular matrix. By reversing cytoplasmic glycolysis and lactate formation and decreasing stromal fibrosis, tamoxifen can enhance T cell immunosurveillance in the tumor microenvironment (TME) and potentially improve chemotherapy delivery to tumors. Tamoxifen inhibits cancer cell interactions with platelets, which decreases platelet activation and the release of TGF-β1, platelet derived growth factor (PDGF), vascular endothelial growth factor (VEGF), interleukin 6 (IL-6) and insulin like growth factor (IGF-1) from platelets into the TME [1,46,47,54].

## 4. 17β-Estradiol, Hypoxia-Inducible Factor (HIF) and Vascular Endothelial Growth Factor (VEGF)

The effect of estrogen on the production of VEGF is comparable to that of hypoxia. This is because the pro-angiogenic effect of 17β-estradiol (E_2_) is mediated by the nuclear translocation of estrogen receptor-α (ER-α) and also HIF-1α translocation. Moreover, the 17β-estradiol stimulation of both HIF-1α and VEGF is dependent on the phosphatidyl inositol-4,5-bisphosphate-3-kinase (PI3K), which is inhibited by anti-estrogens such as fulvestrant. The molecular interdependence of estrogen and hypoxia has been demonstrated in both cancer cells and human endothelial cells [60]. Recruitment of a malignant tumor stroma via MMT, VEGF and neoangiogenesis is fundamental to the survival and proliferation of epithelial cancers, and up to 80% of such solid tumors are comprised of CAFs [61]. 

### 4.1. Estrogen Receptors (ER-β) and HIF-1β

HIF-1β or Aryl hydrocarbon receptor nuclear translocator (ARNT) is constitutively expressed in most cells, but there is evidence that HIF-1α upregulates HIF-1β expression under hypoxia in some cancers. This indicates a potential HIF-1α mediated feed-forward loop of augmented HIF signaling [62]. Conversely, HIF-1α activity is inhibited by estrogen receptor-β (ER-β), which degrades ARNT by ubiquitination. This results in a decrease in the active HIF-1α/ARNT complex. ER-β also attenuates the hypoxic generation of VEGF mRNA by directly decreasing the binding of HIF-1α to the VEGF gene promoter [63]. 

### 4.2. 17β-Estradiol (E_2_) in the Tumor Microenvironment

There is evidence of ER and aromatase expression in stromal and immune cells within the tissue or tumor microenvironment, for example in endometriosis and ovarian cancer, but also gastric and colorectal cancer. Such cells include CAFs, myeloid derived suppressor cells (MDSC), dendritic cells, and transformed mesothelial cells. E_2_ inhibits both cell mediated and humoral immunity by activating ER-α on T-cells, B-cells and non killer (NK) cells, with ER-α46 being the predominant isoform. E_2_ promotes a protumor, immunosuppressive TME by increasing Treg and MDSC populations, tumor cell programmed death ligand (PD-L1) expression, and the inhibition of CD8+ T cell and NK cell induced apoptosis. E_2_ decreases Th1 activity by promoting cytokine production (IL-6, IL-4, TNFα, IL-17A), and M2 TAM infiltration. The paracrine secretion of E_2_ and IL-6 by CAFS also contributes. This may explain the benefits of estrogen depletion in estrogen insensitive tumors, including A7C11 mammary tumor cells, B16 melanoma cells and intraperitoneal Lewis lung carcinoma. Combining estrogen receptor blockade and PD-L1 inhibition may be synergistic and independent of the sensitivity of tumor cells to estrogen signaling [64,65]. 

## 5. TGF, Platelets, Podoplanin Promote Tumor Progression

Both tumor cells and CAFs overexpress podoplanin, which is a type I transmembrane mucin-like sialoglycoprotein. Podoplanin, also known as *Aggrus*, has a major role in platelet aggregation and metastasis formation through the binding to its platelet receptor, C-type lectin-like receptor 2 (CLEC-2) [66]. Podoplanin mediated tumor cell-platelet aggregation promotes the release of platelet derived TGF-β1, which is critical for EMT, invasion and metastasis. By blocking TGF-β1 signaling via pan-TGF-β neutralizing mAb 1D11 or TGF-β receptor inhibitors (galunisertib, targets both TGFβR1 and TGFβR2; SB431542, targets TGFβR1), tumor cell invasiveness and EMT can be abolished. The use of sunitinib inhibiting PDGF receptor did not have any effect on podoplanin induced EMT. Release of PDGF and TGF-β during podoplanin-positive tumor cell-induced platelet aggregation is suppressed by anti-podoplanin mAbs. Increased podoplanin expression in CAFs and a positive correlation between CAF podoplanin expression and poor prognosis were demonstrated in lung adenocarcinoma, the invasive ductal carcinoma of breast and pancreas, and melanoma [67,68]. Release of platelet derived TGF-β1, FGF2 and EGF also contributes to increased podoplanin expression. This highlights the role of platelets and podoplanin in promoting tumor progression via MMT, which is similar to the enhanced fibrin deposition, ECM formation and podoplanin expression observed in the induction and progression of EPS [66]. 

A combination of a TGF-β1 inhibitor and cisplatin in ovarian cancer cell lines had a stronger anti-proliferative effect than the additive effects of each treatment alone [69]. The inhibition of thrombin by dabigatran synergistically enhanced the effect of cisplatin in ovarian cancer cell growth and ascites in vivo. This was thought to be related, in part, to the decreased production of IL-10, IL-6, TGF-β and VEGF, and the alleviation of the tumor immunosuppressive microenvironment. Dabigatran etexilate treatment, with or without cisplatin, reduced platelet activation and inhibited the generation of tissue factor (TF) positive microparticles in the circulation in a murine ID8-luc ovarian cancer model [70]. Dabigatran blocks the thrombin and fibrinogen-induced differentiation of normal fibroblasts into myofibroblasts and decreases pSMAD3 (8.1 fold), TGF-β1 (1.8 fold), CTGF (10.5 fold), platelet-derived growth factor-AA (PDGF-AA) release, as well as the expression of proinflammatory chemokines and ECM proteins, such as α-SMA, collagen, fibronectin and TNC [71]. 

### 5.1. Tamoxifen and Tumor Cell Platelet Activation

Activated platelets coat tumor cells with a cell-fibrin-platelet aggregate shield, protecting them from shear forces and host immune attack by NK lymphocytes [66]. The transference of major histocompatibility class (MHC) I proteins and glucocorticoid-induced tumor necrosis factor receptor-related (GITR) ligand from platelets to tumor cells (trogocytosis) also assists in the tumor evasion of host immunosurveillance. Trogocytosis disrupts the recognition of tumor cell missing self, resulting in impaired cytotoxicity and IFN-γ production by NK cells [72,73]. Platelet-derived TGF-β, released upon tumor cell-platelet interaction, also inhibits NK cell mediated immunosurveillance through the downregulation of the activating NK receptor NKG2D [66]. The formation of ovarian cancer spheres, chemotaxis, cancer cell migration, EMT and stem cell markers are increased by platelets. This enhances both haematogenous and transcoelomic metastasis [74,75]. 

Tamoxifen has been shown to inhibit tumor cell-platelet activation. Human platelets contain both glycosylated estrogen receptor α and β. However, the effects of tamoxifen on platelet inhibition are not mediated directly through platelet estrogen receptors. Tamoxifen inhibits the protein kinase C (PKC) pathway via phospholipase C gamma 2 (PLCγ2), as well as the p38 MAPK pathway in platelets. Tamoxifen pretreatment *ex vivo* of normal platelets inhibited the release of platelet derived VEGF, IGF-1 and IL-6 and completely prevented platelets from promoting the transendothelial migration of breast cancer cells. Moreover, 4-hydroxy tamoxifen was a potent inhibitor of platelet aggregation and protein release, including angiogenin, CCL5 (RANTES), CXCL1, EGF, MMP-1, TIMP, PDGF and TGFβ. Furthermore, 4-OH tamoxifen increased the release of angiopoietin-1, a protein known to inhibit tumor angiogenesis. Platelets from breast cancer patients who were being treated with tamoxifen showed significant inhibition of aggregation when exposed to breast cancer cells or adenosine diphosphate (ADP), but not to thrombin receptor activating peptide (TRAP). Tamoxifen thus appears to directly inhibit cancer cell-platelet aggregation and platelet stimulated EMT in an in vitro breast cancer cell model. This has important implications for the potential use of tamoxifen as an antiplatelet and antiproliferative agent in both estrogen dependent and estrogen “independent” cancers, such as gastric, pancreatic and colorectal peritoneal metastasis [76,77]. 

### 5.2. Tamoxifen and Megakaryocytes

An additional effect of tamoxifen is the antagonism of β-estrogen receptors in megakaryocytes. Tamoxifen was shown in vivo to decrease platelet counts by 45% [78]. Platelet production is regulated by thrombopoietin (TPO), transcription factor GATA-1, nuclear factor erythroid-derived 2 (NF-E2) and ER-β [79]. Bone marrow megakaryocytes in both male and female humans and mice produce 17β-estradiol and also express ER-β and androgen receptors (AR) [80]. Estrogen- and androgen-responsive genes, such as nitric oxide synthase (an inhibitor of platelet aggregation), superoxide dismutase, gp130, and thromboxane A2, are present in megakaryocytes and/or platelets. Megakaryocyte estradiol synthesis is upregulated by the megakaryocyte/erythrocyte specific transcription factor p45 NF-E2. NF-E2 is the gene for 3β-hydroxysteroid dehydrogenase (3β-HSD), which is essential for the formation of 17β-estradiol [81]. Autocrine megakaryocyte 17β-estradiol secretion triggers the last stage of megakaryocyte maturation involving proplatelet formation (PPF), resulting in the release of platelets. The addition of exogenous extracellular 17β-estradiol also stimulates PPF (by >50%) [76,82]. This means that a TPO independent, positive feedback loop of megakaryocyte platelet production exists, involving a 17β-estradiol/ER-β/GATA1/NF-E2/3β-HSD/17β-estradiol pathway [79]. 

Preoperative thrombocytosis is a significant risk factor for tumor progression and poorer survival in ovarian, pancreatic, colorectal, hepatocellular, gastric and esophageal cancers [83,84]. Thrombocytosis is, in part, related to the circulating IL-6 stimulation of human hepatocyte IL-6 receptors to trigger TPO production and megakaryocyte activity. IL-6 signaling is also able to induce VEGF receptor (VEGFR2) expression in colorectal cancer (CRC) cells. This produces a feed forward circuit in colorectal carcinogenesis which includes IL-6, TPO, platelets and VEGF [84]. SMAD4 mutations in CRC may result in dysfunctional TGF-β1 signaling and increased IL-6 production [84]. Loss of SMAD4 expression is associated with poor prognosis in resected stage II, III and IV CRC [85]. 

### 5.3. Tamoxifen, Thrombocytosis, and Cancer

Thrombocytosis (Plts > 450,000/μL) is a predictor of refractory response to anti-VEGF treatment (bevacizumab, sorafenib, sunitinib) or taxane-based chemotherapy when compared to patients with normal platelet counts [83,84]. Decreasing the platelet count by antiplatelet antibodies (APA) in a murine ovarian cancer model lowered tumor mass by 65% (*p* = 0.008), which was equivalent to the effect of docetaxel treatment (70%). Using docetaxel and APA resulted in an additional 62% reduction in aggregate tumor weight compared to docetaxel alone (*p* = 0.04). Mice given platelet transfusions had a 70% increase in mean aggregate ovarian tumor mass compared to untreated controls in a serous ovarian adenocarcinoma (SKOV-ip1) model [83]. The TPO independent tamoxifen blockade of β-estrogen receptors in megakaryocytes and the lowering of circulating platelets may be an additional mechanism for the antiproliferative effect of tamoxifen [78,86]. However, the increased risk of VTE in female patients treated with tamoxifen is thought to be related to increased ROS generation in platelets due to tamoxifen, mainly via superoxide increasing platelet aggregation [87]. 

### 5.4. Tamoxifen and HSP90

HSP90 is essential as a chaperone molecule for ER-α hormone binding, dimer formation, and binding to the nuclear estrogen-response elements (ERE) [88]. Elevated HSP90 has been shown to promote fibroblast activation by stabilizing the TGF-β receptor and integrin linked kinase (ILK), and enhancing chemokine receptors (CD44, CCR5, hyaluronan ligand) [89]. The high dose inhibition of HSP90 causes the activation of the stress-responsive transcription factor heat-shock factor 1 (HSF1) and the release of HSP70, leading to acquired tamoxifen resistance in ER positive breast cancer. However, low dose HSP90 inhibition with ganetespib (<10 nmol), a synthetic second generation HSP90 inhibitor, or low dose administration of the naturally occurring HSP90 inhibitor geldanamycin, did not inhibit cell growth alone, but profoundly decreased the emergence of tamoxifen resistant breast cancer cell clones. This resulted in dramatic improvements in event free survival in a murine in vivo breast cancer study. Improved differentiation, expression of the normal epithelial marker MUC1 and profound cell cycle arrest in breast cancer xenografts were also observed. This was associated with substantial and uniform reduction in levels of the cellular proliferation proteins cyclin-dependent kinase 4 (CDK4), Cyclin D1, and myelocytomatosis (MYC). The molecular mechanism of combined ER antagonism and low dose HSP90 inhibition was thought to be a blockade of ER and HER2 upregulation induced by tamoxifen alone. This may be a useful strategy in the prevention of EPS, PARP induction and chemoresistance during intra-peritoneal chemotherapy [90]. 

### 5.5. Tamoxifen and Different Estrogen Receptors (ER-α, ER-β, GPER-1)

**Figure 4:**
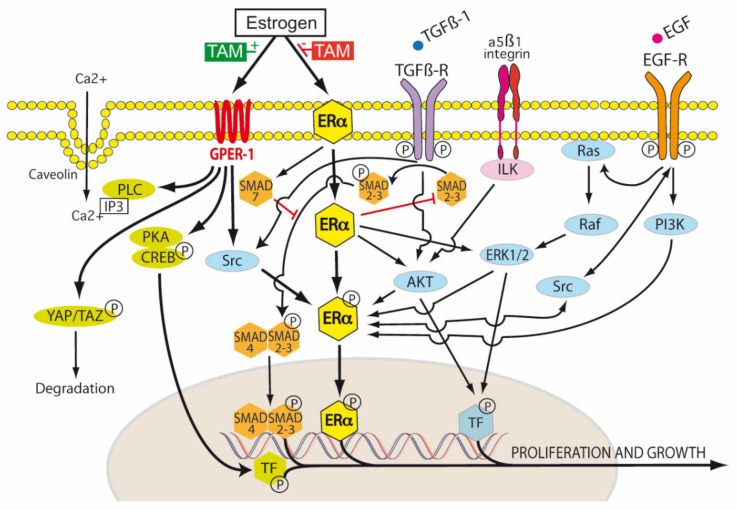
Tamoxifen modulation of membrane and nuclear estrogen receptor (ER) signaling. Transduction of the extracellular environment occurs at the cell membrane. This is mediated by transforming growth factor-β1 receptor (TGFβ-R), integrin linked kinase (ILK), caveolin ion channels and membrane bound receptor tyrosine kinases (RTKs), for example, epidermal growth factor receptor (EGFR). In the TGF-β1 canonical pathway, TGF-β1 first binds to TβR-II, and then the recruitment of TβR-I occurs. Both receptors then form the TβR complex, in which TβR-II phosphorylates and activates TβR-I. The active TβR-I then phosphorylates SMAD2/3. This results in the cytoplasmic retention of p-SMAD2/3 and association with SMAD4. The resulting SMAD complex is translocated to the nucleus and activates SMAD-dependent transcriptional activity. SMAD7 decreases the stability and activity of TβR-I by inducing receptor degradation. SMAD7 also competes with SMAD4 to associate with SMAD 2/3. Tamoxifen blocks canonical TGF-β1 signaling by inhibiting SMAD2/3 and promoting SMAD7 (red blockade arrows). The activation of the non-receptor tyrosine kinase Src occurs via the phosphorylation of membrane bound RTKs, including estrogen receptor alpha (ER-α). There is a reciprocal phosphorylation of Src, ER-α and RTKs—this is dependent, in part, on the tissue levels of tamoxifen and the expression levels of membrane ER-α. Estrogen, tamoxifen and fulvestrant are *agonists* of G protein-coupled estrogen receptor-1 (GPER-1). Estrogen regulates Hippo signaling via GPER in breast cancer by phospholipase C (PLC), protein kinase C (PKC), Yes associated protein (YAP), and transcriptional coactivator with PDZ-binding motif (TAZ). In ER-α negative breast cancers, GPER activation turns *on* the Hippo pathway, causing the phosphorylation of YAP and the cytoplasmic retention of pYAP, which is then marked for proteosomal degradation. However, when the Hippo pathway is turned *off* (for example by aberrant Src activation), unphosphorylated YAP is able to be translocated to the nucleus and activate transcription targets. Estrogen receptors also influence non-canonical TGF-β1 signaling via PI3K (phosphatidylinositol 3-kinase) and Akt (Protein Kinase B) pathways. Tamoxifen can act as an ER-α antagonist in some tissues (breast) and an ER-α agonist in others (endometrium, osteoblasts, endothelium), hence its pleiotropic effects. It is thus classified as a selective estrogen receptor modulator (SERM) [1,47].

ER-α exists in three isoforms: ER-α66, ER-α46 and ER-α36 [91]. The ER-α66 isoform is the traditional ligand-dependent nuclear transcription factor, regulating gene expression by binding estrogen response elements in DNA. This results in the upregulation of Bcl-2, cyclin D1, and the insulin-like growth factor 1 (IGF-1) receptor. ER-α46 lacks the nuclear transcription activation domain AF-1, but can still bind the ERE. It mediates rapid estrogen signaling, including activation of the Src/PI3K/AKT pathway. ER-α36 lacks both AF-1 and AF-2 domains, but retains the ability to bind DNA, dimerize and activate ligand binding pathways. These include extranuclear sites of ER-α36 cytoplasmic and plasma membrane signaling involving cytosolic kinases, again with rapid activation and signaling. ER-α36, in the presence of low levels of 17β-estradiol, induced the phosphorylation of c-Src at Tyr416 and the dephosphorylation of c-Src at Tyr527 in gastric cancer cell lines expressing ER-α36. This activated p-Src/cyclin D pathway has been shown to be associated with tumor proliferation, invasion and lymph node metastasis in patients with gastric cancer [91] (Figure 4).

There are very low or absent levels of ER-α66 in gastric cancer with high expression of ER-α36; ER-β expression may be protective against invasiveness [91]. Tang et al. (2017) showed that ER-α expression was an independent risk factor for decreased overall survival in resected gastric cancer patients. Lentiviral vector expressing shRNA targeting ER-α suppressed the proliferation, migration and invasion of gastric cancer cells in vitro, by upregulating the expression of p53, p21, p27, and E-cadherin, and inhibiting cyclin D1 expression [92]. Loss of the cell-cell adhesion molecule E-cadherin is fundamental to EMT, carcinogenesis, invasion and metastasis, and in ovarian and breast cancer, is regulated by ER-α interactions with SNAIL and SLUG. Mutation or loss of E-cadherin (CDH1) is found in both familial and sporadic forms of diffuse gastric cancer [93], and 17β-estradiol also down-regulates E-cadherin through ER-α in these tumors. ER-α expression is more common in poorly differentiated and signet ring cell gastric carcinoma (Lauren diffuse-type), than in well or moderately differentiated gastric carcinoma (Lauren intestinal-type). The use of fulvestrant, an analogue of E_2_ that down-regulates and degrades ER-α without agonism (SERD), has been shown to suppress E_2_-induced proliferation of ER-α positive gastric cancer cells, and synergize with paclitaxel. This is because E_2_ enhances cell motility by destabilizing microtubules via the deacetylation of α-tubulin, which leads to paclitaxel resistance [94]. 

**Figure 5:**
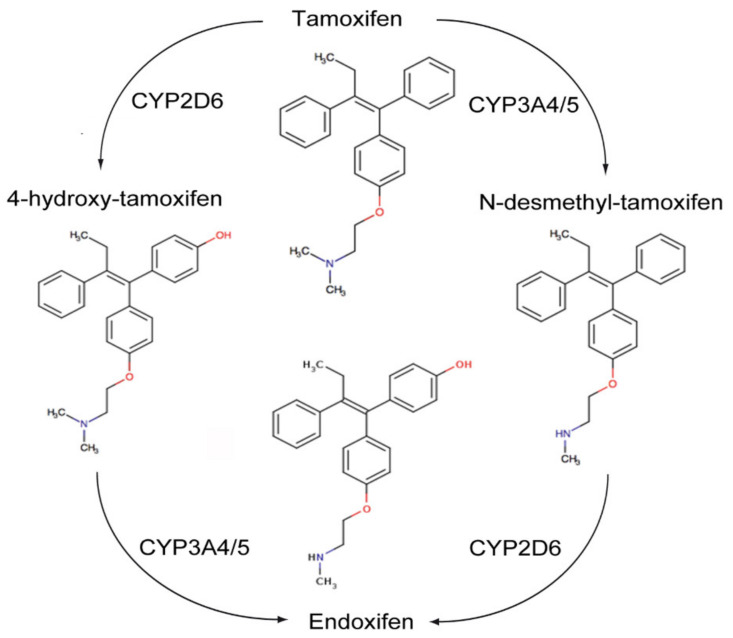
Metabolism of Tamoxifen.

The transactivation of ER-α can occur by the classic ERE/ER-α66 pathway, or by non-classical transactivation where other transcription factors, including the Fos-Jun complex, NF-κB and specificity protein 1 (Sp-1), are responsive to ER activity [95]. Tamoxifen can have antagonist activity for ER-α via AF-2, or partial agonist activity for ER-α in endothelium, bone osteoblasts or endometrium via AF-1 [95]. Tamoxifen is metabolized by hepatic microsomal cytochrome P450 (CYP) enzymes to 4-hydroxy tamoxifen and N-desmethyltamoxifen (NDT). 4-hydroxy tamoxifen has an ER-α binding affinity more than 100 times that of parent tamoxifen, which explains the superior in vivo activity of tamoxifen compared to its in vitro activity (Figure 5). Furthermore, 4-hydroxy tamoxifen binding to ER-α causes a different conformational change as compared to 17β-estradiol ligand binding, as the groove that interacts with the steroid receptor coactivator-1 (SRC-1) remains covered by the twelfth α helix of the ligand binding domain (LBD) [95]. Thus, 4-OHT predominantly antagonizes ER-α activation, inhibiting cellular proliferation and promoting apoptosis in cancers with high levels of ER-α expression. 

ER-β has opposing effects to the ER-α on cellular proliferation, apoptosis and migration. Tamoxifen and its metabolites also modulate ER-β. This is dependent on relative ER-β tissue levels and polymorphisms in several CYP enzymes. For example, endoxifen stabilizes ER-β, promoting ER-α/β heterodimer formation, and has increased inhibitory effects on the expression of ER-α target genes when ER-β is expressed. Endoxifen is the most potent tamoxifen metabolite in the inhibition of ER-α. However, there are highly variable steady state endoxifen plasma concentrations (range 5–180 nM) in women receiving standard tamoxifen therapy (20 mg/day). This is related to variations in the CYP2D6 mediated oxidation of NDT to endoxifen in individual patients [96,97,98] (Figure 5). The tumor suppressant role of ER-β has been demonstrated in ovarian cancer, CRC and mesothelioma. Metastatic ovarian cancer is often characterized by the complete loss of ER-β expression [99,100]. 

The third ER type is G-protein-coupled estrogen receptor (GPR30 or GPER-1), for which tamoxifen is an agonist rather than an antagonist. GPER-1 signals to phospholipase, which releases inositol triphosphate, and to adenylyl cyclase, which produces cAMP. cAMP is involved in the up-regulation of glycolysis, which also generates dicarbonyl side products such as methylglyoxal. GPER-1 receptor activation is associated with acquired tamoxifen resistance in breast cancer patients, which may be correlated with decreased sensitivity to GDP/AGE related oxidative stress [101]. The activation of GPER-1 by tamoxifen is also associated with an increase in risk of endometrial cancer (1.5–6.9 fold) or endometriosis in post menopausal women with breast cancer treated with long term tamoxifen [102]. 

However, in PDAC, which is characterized by an hypoxic, poorly vascularized, desmoplastic stroma, the activation of GPER-1 by tamoxifen resulted in a 4-fold decrease in GLUT levels and a 70% decrease in HIF-1α. Pancreatic stellate cells (PSC) were shown to express ER-α, ER-β and GPER-1, but only the agonism of GPER-1 by tamoxifen was shown to de-activate PSCs. Tamoxifen was found to remodel the dense PDAC stroma by its downregulation of more than 25 hypoxia related genes and up-regulation of more than 30 revascularization genes in pancreatic stellate cells. Via GPER-1 dependent signaling, tamoxifen decreased type I fibrillar collagen, vimentin, α-SMA and fibronectin synthesis, lysyl oxidase (LOX)-L2 levels, myosin dependent contractility and MMP-2 expression in PSCs. Total Yes-associated protein (YAP) levels and YAP nuclear translocation in PSCs were reduced by 33% and 50% respectively by tamoxifen. This resulted in inhibition of the YAP target genes connective tissue growth factor (CTGF) and ankyrin repeat domain 1 (ANRKD1). The activation of the YAP pathway is thought to be important in the progression of PDAC precursor lesions (PanIN) into KRAS dependent invasive PDAC. HIF-1α increases the nuclear translocation of unphosphorylated YAP to the nucleus via Ras homolog family member A (Rho-A). The nuclear translocation of unphosphorylated YAP also enhances HIF-1α induced upregulation of the Pyruvate kinase muscle isoenzyme 2 gene (*PKM2*). PKM2 is important in switching mitochondrial oxidative phosphorylation to cytoplasmic glycolysis and the production of lactate (Warburg effect). YAP is phosphorylated by the tamoxifen/GPER activation of the Hippo pathway, which results in cytoplasmic retention of pYAP and its proteosomal degradation (Figure 4). Tamoxifen was also shown to decrease proliferation and increase apoptosis in the epithelial cells of PDAC, which was mediated via the cell surface GPER-1 mechanical regulation of HIF-1α, a mechanism independent of hypoxia. The ability of PSCs to stiffen their cytoskeleton and contract the ECM in response to external forces applied to integrin receptors was impaired by tamoxifen. PDAC cancer cell invasion of the ECM due to PSC stromal remodeling was abolished by tamoxifen. The infiltration of PDAC tissues by M2 polarized TAMs was also reduced by tamoxifen. M2 TAMs are associated with an immunotolerant TME and ECM secretion and remodeling in PDAC. Thus, tamoxifen may inhibit PDAC growth, but also enable better chemotherapy drug delivery to PDAC by inhibiting the myofibroblastic differentiation of PSCs, reducing stromal stiffness and extracellular matrix invasion and improving vascularity [103,104,105,106] (Figure 4). 

Tamoxifen also has activity against cancers that have low levels of ER-α (BT-20 breast cancer) or different ER-α isoform expression (gastric cancer). In gastric cancer cells with ER-α36 expression, tamoxifen inhibited in vitro proliferation of gastric cancer cells in a time and dose dependent fashion. Because a functioning Src axis is required for ER-α coregulator mediated extranuclear actions, a combination of tamoxifen and a Src kinase inhibitor (dasatinib) may be synergistic in ER-α/HER2 expressing tumors, including gastric cancer and tamoxifen resistant breast cancer [100,107]. Tamoxifen resistance can be related to the increased phosphorylation of p130Cas/BCAR1, FAK and Src, which is mediated by membrane integrin based adhesion signaling [108]. Low dose anti-estrogens (4-OH tamoxifen, fulvestrant) in ER-α66 negative breast cancer with high expression of ER-α36 were shown to activate Src and MAPK/ERK by phosphorylation and activate Cyclin D1 via the Src/EGFR/STAT5 pathway. However, high dose anti-estrogens caused the inactivation of Src by phosphorylation at Y527 and did not stimulate downstream Src signaling pathways [109].

Tamoxifen also has killing effects that are independent of ER-α expression (for example, ovarian A2780 cancer, T cell leukemia, hepatoblastoma cells) [110,111]. 

## 6. Estrogen Independent Effects of Tamoxifen

Such ER-α independent mechanisms include the induction of c-myc expression, modulation of TGF-β1 expression, and the generation of oxidative stress. Oxidative stress leads to oxidation of membrane lipids, production of ceramides and 4-hydroxynonenal (4HNE), JNK phosphorylation and breast cancer cell apoptosis. Ceramides accumulate after cell death from radiotherapy or chemotherapy, and are lipid-signaling mediators that activate caspase-3. This leads to the cleavage of PARP and other proteins resulting in apoptosis. Tamoxifen increases PARP cleavage through both caspase-3 dependent and independent mechanisms. However, tamoxifen pretreated breast cancers in cell culture and murine models demonstrated subsequent adaptation to tamoxifen induced oxidative stress. Increased expression of the downstream transcription targets of the antioxidant response element (ARE) were demonstrated, including Nrf2, antioxidant genes (NQO1, HMOX1, SOD1) and the multidrug resistance transporters (MDRT: ABCC1, ABCG2, ABCC3). ABCC1 transports the toxic oxidation product 4HNE out of cancer cells. P-glycoprotein mediated MDRT also increases the efflux of cytotoxic drugs such as anthracyclines, vinca alkaloids, colchicines, etoposide, and paclitaxel, and RTK inhibitors (imatinib, nilotinib, dasatanib, bosutinib), which reduces their treatment efficacy [112,113]. High Nrf2 and ABCC3 expression in tamoxifen treated ER-α positive breast cancers patients was associated with poor prognosis for treatments with transtuzumab, anthracyclines and taxanes [110]. Recently, it was shown that low dose aspirin was able to restore tamoxifen sensitivity in ER-α positive, tamoxifen resistant breast cancer cells. This was thought to be mediated by aspirin combined with tamoxifen downregulating cyclinD1 and blocking the cell cycle in the G0/G1 phase [114]. 

### 6.1. Tamoxifen and RAGE

The Warburg effect leads to oxidative stress, increased glycolysis, the production of reactive aldehydes (methylglyoxal) and the insufficient degradation of glycated proteins in cancer cells, resulting in accumulation of AGEs (hydroimidazolone and argpyrimidine). Spontaneous degradation in tumor cells of the triose phosphate intermediates of glycolysis, dihydroxyacetone phosphate and glyceraldehyde 3-phosphate, is the mechanism of formation of methylglyoxal [115]. The receptor for advanced glycation endproducts expression is mediated by the interaction of HIF-1 with an HRE sequence on the RAGE promoter, stimulated by hypoxia or ischaemia [116]. In EPS, AGE-RAGE promotes PMC transitioning to a mesenchymal phenotype, and in the case of peritoneal malignancy, a transformed malignant stroma [117]. The metabolic switch to aerobic glycolysis in cancers promotes a perpetual effect of AGE production, RAGE expression and ligand-RAGE-NF-κB signaling, which is the major molecular link between hyperglycemia, glycation, inflammation, hypoxia, oxidative stress, and carcinogenesis [118]. The binding of RAGE to oncogenic KRAS facilitates HIF-1α activation and promotes pancreatic tumor growth under hypoxic conditions [119]. High levels of RAGE expression are significantly associated with the histological grade, nodal status, metastasis status, and AJCC stage in gastric cancer, similar to previous findings in oral squamous cell and non-small cell lung cancer (NSCLC). RAGE is also related to poor prognosis in ovarian, CRC and hepatocellular carcinoma [118]. The interaction of AGE-RAGE leads to the activation of NF-κB, as well as MEK and MAP kinase pathways [120]. Dicarbonyl stress due to methylglyoxal adducts was directly correlated with depth of tumor invasion in CRC [115]. AGE, TNF-α, and 17β-estradiol specifically up-regulate RAGE mRNA and protein levels in human vascular endothelial cells (HMVEC). This is mediated by two distinct nuclear complexes, p65/p50 NF-κB and Sp-1/ERα. Moreover, 4-OH tamoxifen completely abolished the E_2_-induced RAGE mRNA induction in HMVEC [121,122]. The stimulation of TGF-β1 by AGE-RAGE interaction is inhibited by tamoxifen, a novel mechanism which may be useful in RAGE overexpressing cancers [118].

#### 6.1.1. Other Systemic Approaches for Treating MMT

Therapeutic strategies to minimize MMT in EPS include direct TGF-β1 blockade, monoclonal anti-RAGE antibodies, SMAD7 transgene expression, BMP-7, BMP-4, rapamycin, anti-CTGF antibodies, microRNA30a, rosiglitazone, or Src inhibition [46,49,50,123,124,125,126]. Oral rapamycin, which inhibits mTOR, has been shown to reduce peritoneal thickening, inflammation, angiogenesis, and lymphangiogenesis by the blockade of HIF-1α, TGF-β1 and VEGF in murine models of PD induced EPS [46]. Other drugs that have been proposed in EPS treatment include mycophenolate mofetil, colchicine, azathioprine, angiotensin converting enzyme inhibitors/angiotensin II receptor blockers, calcitriol, benfotiamine, thalidomide and dipyramidole [46,58,127,128,129,130]. The use of non-steroidal immunosuppressants (azathioprine, mycophenolate mofetil, rapamycin), with or without glucocorticoids, may improve EPS, but there are no clinical trials and effectiveness is based on case reports [124]. Colchicine, pentoxifylline, N-acetylcysteine, rosiglitazone (PPAR agonist), pirfenidone, thalidomide and itraconazole have been studied in murine EPS models and can be categorized as experimental agents at present [129].

#### 6.1.2. Targeting the TGF-β1 Pathway 

Bi-directional communication between mesothelial cell derived CAFs and cancer cells via TGF-β1/HIF mediated MMT creates an environmental niche for the progression of peritoneal metastasis. This includes a pseudohypoxic microenvironment for lactate and glutamine shuttling between CAFs and cancer cells (reverse Warburg effect) [131,132], chemotherapy resistance [133,134], immunotolerance via Treg cells [135], and positive feedback loops involving IL-1/TGF-β1/VEGF/ROS and HIF [136]. Ovarian and colorectal peritoneal cancer cells show an increased production of TGF-β1 but less nuclear translocation of SMAD3 compared to MMT derived CAFs [136]. In vitro and in vivo blockade of the TGF-β1-pSMAD3 pathway results in less fibrosis, reversal of MMT, diminished peritoneal metastatic progression and improved survival [136]. Strategies for TGF-β1 blockade include neutralizing TGF-β antibodies, anti-sense TGF oligodeoxynucleotides [137], engineered soluble TβR-II antagonists [138], small molecule TGF-βRI kinase inhibitors [139], and Src inhibition with dasatanib or KX2–391 [125,140]. Galunisertib may be synergistic with anti-PD-L1 checkpoint inhibitors (e.g., nivolumab, durvalumab) in blocking the TGF-β1 suppression of host immune surveillance by Treg cells in the tumor microenvironment. Galunisertib was effective in mouse pancreatic cancer models in inhibiting TGF-β1-mediated EMT and cancer cell migration, and inhibiting pSMAD2/3 induced proliferation of stromal fibroblasts. However, it did not prevent bFGF or PDGF stimulated fibroblast proliferation. Galunisertib monotherapy effectively inhibited tumor growth in a number of human cancer xenografts, and was synergistic with 5-FU and paclitaxel in in vitro gastric cancer cell studies, and with sorafenib in HCC cell lines [141,142,143,144,145]. The inhibition of core fucosylation of Activin receptor-like kinase-5 (ALK-5), TBRII and PDGF receptor by Fut8 small interfering RNA (siRNA) successfully suppresses the activation of TGF-β1/ALK-5/SMAD2/3 and PDGF/ERK signaling pathways and attenuates rat PMC EMT in vitro [146]. This has important implications for the control of GDP/AGE induced CAPD EPS, but also glycosylation related renal interstitial fibrosis and carcinogenesis [132,147,148,149].

#### 6.1.3. Src Inhibition 

The inhibition of Src with KX2-391 (a highly selective Src inhibitor) or siRNA resulted in the attenuation of peritoneal fibrosis in a chlorhexidine gluconate model of EPS. KX2-391 inhibited the phosphorylation of Src, which resulted in the suppression of both the TGF-β1/pSMAD3 and PI3K/Akt signaling pathways and the prevention of MMT in human PMCs [125]. Src can be activated by multiple transmembrane receptor kinases (RTKs), including FGFR, EGFR, human epidermal growth factor receptor 2 (HER2 or ErbB2), PDGFR, insulin-like growth factor-1 receptor (IGF-1R) and c-Met/hepatocyte growth factor receptor (HGFR), as well as other membrane receptors, such as ER-α, integrins and erythropoietin receptor. c-Src can phosphorylate ER-α on tyrosine537, leading to potential 2 way crosstalk and positive feedback loops in tissues expressing ER-α [109,150,151]. The addition of a Src inhibitor (dasatanib) has been shown to reverse resistance to RTK inhibitors, such as the FGFR3 inhibitors infigratinib and erdafitinib [152].

#### 6.1.4. Connective Tissue Growth Factor Inhibition 

A further potential target in EPS is CTGF or CCN2. CTGF is an extracellular matrix-associated heparin-binding protein. CTGF is a downstream target of TGF-β1 and YAP and activates TGF-β-mediated fibrosis [153]. It is also downregulated by tamoxifen [54]. CTGF has a pivotal function in cell adhesion, migration and proliferation. It is a mediator of angiogenesis and tissue wound repair, and is critically involved in fibrotic disease and several forms of cancer, including PDAC, gastric and CMS4 colorectal cancer [154]. CTGF is highly expressed in EPS, and regulated (besides TGF-β1) by the pro-fibrotic molecules angiotensin II and endothelin-1 [155]. CTGF monoclonal antibody blockade suppressed TGF-β1 induced fibroblast proliferation and myofibroblast differentiation, PMC MMT and VEGF-A production in a chlorhexidine gluconate murine model of EPS [155]. Recently, CCN3 (or nephroblastoma over expressed gene (NOV)) was shown to completely block renal cortex mRNA expression of all four key fibrosis markers (CTGF, Collagen 1a2, TGF-β1, and PAI-1), in a rat model of diabetic nephrosclerosis and mesangial cell proliferation [153]. 

#### 6.1.5. Leptin Inhibition in EPS

Another potential approach in EPS is the inhibition of Leptin, a downstream target of stabilized HIF. Leptin is released from peritoneal adipocytes in response to glucose containing PD fluid [54]. Leptin is a pro-inflammatory adipocytokine which can increase TGF-β1 synthesis and synergize the TGF-β1 induced conversion of peritoneal mesothelial cells into myofibroblasts. 

#### 6.1.6. Glucocorticosteroids (GC)

There are only limited observational studies and case reports describing the effect of high dose glucocorticosteroids in the management of EPS. The use of concurrent GC may have additive effects with tamoxifen, as the hydrocortisone blockade of Interleukin-6/TGF-β1 induced peritoneal MMT is associated with the inhibition of Erk1/2 [156]. Starting glucocorticoids early in the course of the disease was more effective in a Japanese study of GC monotherapy in 79 patients with EPS. The return of intestinal function was achieved in 79% of stage II EPS patients (mild inflammation, nausea and diarrhoea, partial intestinal encapsulation), 57% of stage III EPS patients (mild to severe inflammation, ileus, major encapsulation, adhesion), and in 50% of stage IV EPS patients (chronic ileus, shrinkage of bowel loops), with associated respective mortality rates of 3.6%, 14.3%, and 25% [129].

#### 6.1.7. Vitamin D Receptor Agonists

The activation of vitamin D receptors has been shown to reduce inflammation and fibrosis in EPS and also acute/chronic pancreatitis [130,157]. This is related to the inhibition of TGF-β1/SMAD signaling by genomic competition, and the blockade of NF-κB activation and p65/p50 nuclear translocation. In PDAC, VDR genomic targets include the extracellular matrix, the sonic hedgehog pathway, cytokines/chemokines such as IL6, growth factors (CTGF) and CXCL12, a mediator of T lymphocyte blockade [157]. The VDR is expressed in PDAC stroma, and when activated, acts as a master transcriptional regulator of PDAC PSCs by reversion to a quiescent state. Calcipotriol is a potent and non-hypercalcemic synthetic vitamin D analogue, which activates VDR specifically in PSCs in pancreatic adenocarcinoma models. Calcipotriol was shown to induce lipid droplet formation in PDAC PSCs, reduce tumor volume and stromal density, improve tumor vascularity, increase intra-tumoral gemcitabine (by 500%), block TGF-β/SMAD signaling and decrease the release of IL-6, CTGF and CXCL12. The inhibition of CTGF can improve the efficacy of gemcitabine in PDAC, and CXCL12 inhibition can improve T cell immunosurveillance in the TME. When combined with gemcitabine, calcipotriol improved the survival of KPC mice by 58% with associated significant (29%) long term survivors. Thus, calcipotriol is a promising agent in directing antiproliferative and pro-apoptotic effects on pancreatic cancer cells via VDR activation in PDAC PSCs [157]. A Phase II pilot trial of nivolumab, nab-paclitaxel, paricalcitol (19-nor-1,25-(OH)_2_-vitamin D_2_), cisplatin and gemcitabine in patients with previously untreated mPDAC showed an 83% ORR, median PFS of 8.17 months and median OS of 15.3 months. This high response rate has encouraged expansion of the trial in 2019 [158].

### 6.2. Intraperitoneal Chemotherapy (IPC)

Treatment resistance to intraperitoneal chemotherapy or the progression of peritoneal metastasis can be related to induction of TGF-β1, HIF, MDRT, PARP activation, immunotolerance, persistent EMT of cancer cells or MMT of PMC [136,159,160,161]. Numerous epithelial cancers that metastasize to the peritoneum have an overexpression of TGF-β1. These cancers may also show lack of response to their secreted TGF-β1, but their transformed stroma are still TGF-β1 responsive, leading to hard peritoneal nodules, with elevated stiffness (elastic modulus), hydrostatic pressures and chemoresistance [9,36,136,162]. 

The effect of tamoxifen on TGF-β1 signaling in CAPD EPS has many parallels with MMT in post IP chemotherapy EPS, and CAF activation in peritoneal metastasis and malignant ascites. There is evidence for the use of tamoxifen in ovarian, fallopian tube and primary serous peritoneal cancers as they have high expression of ER-α [99,100], but also in ER-α negative peritoneal cancers. For example, in heavily pre-treated cisplatin resistant ovarian, fallopian and primary peritoneal cancers, the progression free survival with tamoxifen was comparable to that of systemic cytotoxic chemotherapy. Patients with ER-α positive tumors *or* ER-α negative tumors experienced long progression free intervals with tamoxifen treatment [163]. The finding that normal PMCs express ER-α and respond to tamoxifen suggests that tamoxifen may be beneficial in other peritoneal malignancies, such as mCRC, gastric cancer, pancreatic cancer and malignant mesothelioma, where PMC derived stromal interactions and ER-α/ER-β expression ratios are also important [68,103,104,105,164]. 

#### 6.2.1. PARP Expression

Normothermic IPC is potentially limited by PARP expression, TGF-β1 related normoxic stabilization of HIF, the activation of downstream targets of the HRE, TGF-β1 related cancer cell EMT, PMC MMT or the development of EPS, which precludes further IPC treatments. PARP cleavage and blockade of TGF-β1 signaling may be achieved by concurrent tamoxifen treatment, even in non ER-α responsive tumors. Tamoxifen inhibits PMC MMT and cancer cell EMT. This may improve responses to intraperitoneal chemotherapy, as a mesenchymal phenotype predicts chemotherapy resistance, tumor invasion and poor survival [159]. Conversion by tamoxifen from a TGF-β1 induced Warburg effect, with high levels of glycolysis, acidosis and lactate production, to oxidative phosphorylation may limit the synergism between CAFs and peritoneal cancer cells [165,166]. Tamoxifen may assist in the improvement of malignant ascites by the inhibition of TGF-β1 induced, VEGF secreting CAF cells [136]. Overcoming the problem of acquired escape mechanisms related to CAFs in the TME may require a broad spectrum RTK inhibition approach, together with AKT/mTOR inhibitors in combination with tamoxifen therapy [167,168]. 

#### 6.2.2. Intraperitoneal Phytoestrogens—Cantrixil

The use of intraperitoneal phytoestrogens which have multiple modes of action may reduce the toxicity of cytotoxic chemotherapy and overcome the problem of cisplatin resistance in peritoneal metastasis. For example, Genistein is a naturally occurring soy phytoestrogen isoflavone which is a full agonist of ER-β and partial agonist of ER-α and GPER-1. It also promotes autophagy and inhibits EGFR, GLUT1, peroxisome proliferator activated receptor (PPAR) and DNA topoisomerase II. Modifying the benzopyran molecule of Genistein to enhance activity, metabolic stability and ligand binding has led to the development of the third generation benzopyran: Cantrixil. Cantrixil has shown broad cytotoxic activity against chemoresistant ovarian cancer stem cell (CD44+ MyD88+) populations in both 2D and 3D in vitro models, as well as potent cytotoxic activity against non-stem like ovarian cancer cells. Mitotic arrest was a feature of Cantrixil being a direct inhibitor of tubulin polymerization, due to its binding to the colchicine binding site on tubulin. Cantrixil modulates both pro-survival and pro-death pathways, resulting in caspase induced apoptosis. Cantrixil significantly enhanced the cytotoxicity of cisplatin in disseminated peritoneal ovarian cancer and was more effective than paclitaxel administered 12 mg/kg weekly [169,170]. A clinical Phase 1 trial of weekly intraperitoneal Cantrixil in patients with recurrent ovarian cancer is being conducted in a multisite study in Australia and the USA. https://clinicaltrials.gov/show/NCT02903771.

#### 6.2.3. Hyperthermic IntraPeritoneal Chemotherapy (HIPEC)

The rationale for hyperthermia use with intraperitoneal chemotherapy relates to the induction of PARP, homologous DNA recombination and chemotherapy resistance at normal body temperature. Patients undergoing Cytoreductive Surgery (CRS)/Hyperthermic Intraperitoneal Chemotherapy (HIPEC), in whom the intraperitoneal temperature remained consistently above 

40 °C during HIPEC, had a better overall survival (OS) and progression free survival (PFS) [171]. PARP1-mediated polyADP-ribosylation (PARylation) acts to repair DNA damage from chemotherapeutic agents such as mitomycin C, cisplatin or doxorubicin. In studies of BRCA competent peritoneal CRC or ovarian cancers treated with HIPEC cisplatin or doxorubicin at 37 °C or 42 °C, it was found that hyperthermia completely blocked cisplatin or doxorubicin mediated PARylation. This effect was similar to the pharmacological response to three different PARP inhibitors (PARPi): pan-PARPi PJ34, PARP1/2 inhibitor or rucaparib. The potentiation of cytotoxicity by hyperthermia or PARPi was not seen in chemotherapeutic agents which do not induce PARylation, including 5-FU or paclitaxel [172,173]. In selected patients with colorectal peritoneal metastases, CRS and HIPEC using oxaliplatin achieved a median survival of 30 months after complete CRS [174]. Thermotolerance is a mechanism by which tumor cells evade hyperthermia and double strand DNA damage, which diminishes the effect of HIPEC. This involves HSP90, a chaperone molecule for ER-α, TGF-β1, EGFR, AKT, MET, Raf-1 kinase, BRCA2 and PARP [175]. The synergistic combination of hyperthermia, PARP inhibitor and a synthetic HSP90 inhibitor (17-Dimethylaminoethylamino-17-demethoxygeldanamycin) markedly improved tumor killing in vitro and in vivo [176,177]. However, the effect of cisplatin and hyperthermia may be antagonistic in some peritoneal malignancies, including gastric cancer (AGS cells) and colorectal cancer (HCT116 + ch2 (MMR-) cells, and may only be effective at temperatures higher than 43 °C in pancreatic cancer cells (T3M4) [176].

## 7. Conclusions

This review has highlighted the long experience with tamoxifen treatment in EPS, chronic fibrosing conditions and gynaecological malignancy, and the possible extrapolation of this experience to gastrointestinal tumors metastasizing to the peritoneum.

Chronic inflammation and cellular transformation are key features of the *TGF-β paradox* and are related to the transduction of environmental cues by cell surface membrane receptors. The inhibition of TGF-β1 release in EPS and peritoneal metastasis has been shown to prevent and also reverse EMT and peritoneal MMT. As summarized in this review, tamoxifen has multiple mechanisms of action, including the inhibition of TGF-β1 targets, ERE and HRE, HIF-α, SNAIL, RAGE, VEGF, MMP, PAI-I, Leptin, PARP, peritoneal fibrosis, platelet activation, cell migration, BM/ECM invasion, and neoangiogenesis.

There is evidence that both gynecological and gastrointestinal peritoneal malignancies respond to SERM/SERD treatment, related to the inhibition or modulation of nuclear and cytosolic ER-α transactivation. With the known presence of estrogen receptors in gastric and colorectal cancer and the substantial role of MMT and TGF in the pathogenesis of PM, this research might have translational significance. For example, in pancreatic cancer, it has been shown that tamoxifen can induce stromal quiescence, TGF-β blockade and inhibit extracellular matrix formation.

We make the hypothesis that the use of tamoxifen, phytoestrogens or other TGF-β1/HIF/TKR blockade may facilitate the effects of intraperitoneal chemotherapy, particularly in peritoneal cancers with high levels of TGF-β1 expression and mesenchymal phenotypes. Such prevention of activated platelets and a transformed stroma has the potential to improve treatment outcomes for peritoneal metastasis originating from serous ovarian or gastrointestinal cancers.

## Abbreviations

α-SMA	alpha smooth muscle actin
ABCC	ATP-binding cassette subfamily C member
ADP	adenosine diphosphate
AGE	advanced glycation end products
Akt	protein kinase B
ALK	activin like kinase
APA	antiplatelet antibodies
ARNT	Aryl hydrocarbon receptor nuclear translocator or HIF-1β
ATP	adenosine triphosphate
BCL-2	B cell lymphoma-2
BMP	bone morphogenetic protein
BRCA	breast cancer susceptibility gene
CAF	cancer associated fibroblast
CAPD	continuous ambulatory peritoneal dialysis
CCL5	chemokine (C-C motif) ligand 5
CCN	Cysteine-rich 61, Connective tissue growth factor, Nephroblastoma overexpressed
CD	cluster of differentiation
CDH1	E-cadherin gene
CDK	cyclin dependent kinase
CLEC-2	C-type lectin-like receptor 2
COX	cyclo-oxygenase
CRC	colorectal cancer
CRS	cytoreductive surgery
CSC	cancer stem cell
CTGF	connective tissue growth factor, CCN2
CXCL	C-X-C chemokine ligand
CXCR4	C-X-C chemokine receptor type 4
DAMP	damage-associated molecular pattern
ECM	extracellular matrix
EGFR	epidermal growth factor receptor
EM	endometriosis
EMT	epithelial mesenchymal transition
EPS	encapsulating peritoneal sclerosis
ER-α	estrogen receptor alpha
ERE	estrogen response element
ERK	extracellular signal-regulated kinase
FAK	focal adhesion kinase
FGF	fibroblast growth factor
FSP-1	fibroblast specific protein-1
GDP	glucose degradation products
GITR	glucocorticoid-induced tumor necrosis factor receptor-related
GLUT	membrane glucose transporter
GPR30	G protein coupled estrogen receptor 30 (GPER)
HA	hyaluronan
HER2	human epidermal growth factor
HGF	hepatocyte growth factor
HIF	hypoxia inducible factor
HIPEC	hyperthermic intraperitoneal chemotherapy
HMGB1	high-mobility group box 1 protein
HRE	hypoxia response element
HSP	heat shock protein
IFN	interferon
IGF-1	insulin-like growth factor-1
IL	interleukin
ILK	integrin linked kinase
JNK	c-Jun N-terminal kinase
KRAS	Kirsten rat sarcoma virus oncogene
LDHA	Lactate dehydrogenase A
LMW	low molecular weight
MAP3K	mitogen-activating protein kinase kinase kinase
MDRT	multidrug resistance transporter
MHC	major histocompatibility complex
miRNA	microRNA
MMP	matrix metalloprotease
MMT	mesothelial mesenchymal transition
mRNA	messenger ribonucleic acid
mTOR	mammalian target of rapamycin
MYD88	myeloid differentiation factor 88
NF-E2	nuclear factor erythroid-derived 2, Nrf2
NF-κB	nuclear factor kappa light chain enhancer of activated B cells
NK	natural killer
NOD	nucleotide-binding oligomerization domain
NOV	nephroblastoma overexpressed gene
PAI-1	plasminogen activator inhibitor-1
PAMP	pathogen-associated molecular pattern
PARP	Poly (ADP-ribose) polymerase
PDF	peritoneal dialysis fluid
p53	tumor suppressor protein 53
PDGF	platelet derived growth factor
PHD	prolyl hydroxylase
PI3K	phosphoinositide 3-kinase
PK	pyruvate kinase
PKM2	pyruvate kinase isoenzyme M2
PLCγ2	phospholipase C gamma 2
PM	peritoneal metastasis
PMC	peritoneal mesothelial cell
PPAR	peroxisome proliferator activated receptor
PPF	proplatelet formation
PRRX-1	Paired Related Homeobox 1
RAGE	receptor for advanced glycosylation end products
ROCK	rho-associated, coiled-coil-containing protein kinase
ROS	reactive oxygen species
RTK	receptor tyrosine kinase
SERD	selective estrogen receptor degrader
SERM	selective estrogen receptor modulator
SLUG	Zinc finger protein SNAI2
SMAD	Small body +mothers against decapentaplegic
SOD	Superoxide dismutase
SNAIL	Zinc finger protein SNAI1
Src	non receptor sarcoma tyrosine kinase
SRC	steroid receptor coactivator
Sp-1	specificity protein 1
STAT3	signal transducer and activator of transcription
TAK-1	TGF-β-activated kinase 1 or MAP3K7
TAM	tumor (tissue) associated macrophages
TβR	transforming growth factor beta receptor
TF	tissue factor
TGF-β1	transforming growth factor-β1
Th	T helper
TKI	tyrosine kinase inhibitor
TIMP	tissue inhibitor of metalloproteases
TLR2	toll-like receptor
TME	tumor microenvironment
TNF-α	tumor necrosis factor alpha
TPO	thrombopoietin
tPA	tissue plasminogen activator
TRAF6	TNF receptor associated factor
Tregs	regulatory T cells
TWIST1	twist basic helix loop helix transcription factor 1
VEGF	vascular endothelial growth factor
VTE	venous thromboembolism
